# Vitronectin in human hepatic tumours contributes to the recruitment of lymphocytes in an *α*v*β*3-independent manner

**DOI:** 10.1038/sj.bjc.6603467

**Published:** 2006-11-07

**Authors:** S Edwards, P F Lalor, C Tuncer, D H Adams

**Affiliations:** 1 Liver Research Group, Department of Medicine, 5th Floor, Institute of Biomedical Research, Wolfson Drive, The Medical School, University of Birmingham, Birmingham B15 2TT, UK

**Keywords:** vitronectin, adhesion, lymphocyte, liver, tumour, migration

## Abstract

The degree of lymphocyte infiltration is a prognostic factor in liver cancer, but to date the mechanisms by which lymphocytes infiltrate into and are retained in hepatic tumours are poorly understood. We hypothesised that the extracellular matrix glycoprotein vitronectin, a major component of the stroma of hepatic tumours, might play a role in the recruitment and retention of tumour-infiltrating lymphocytes (TIL). Thus, we investigated the ability of vitronectin to support migration and adhesion of TIL isolated from hepatocellular carcinoma and colorectal hepatic metastases. Soluble vitronectin-induced dose-dependent migration of TIL in *in vitro* chemotaxis and haptotaxis assays and vitronectin in tissue sections was able to support TIL adhesion to tumour stroma. Neither adhesion nor migration was inhibited by a function blocking mAb against the major vitronectin receptor *α*v*β*3 and we were unable to detect *α*v*β*3 on TIL *in vitro* or *in vivo* on tumour tissue. However, TIL did express high levels of urokinase-type plasminogen activator receptor (uPAR) and inhibitory antibodies and amiloride both significantly inhibited TIL adhesion to vitronectin and reduced transendothelial migration of lymphocytes across liver endothelium *in vitro*. Thus, we provide evidence that vitronectin in liver tumours can support the recruitment and retention of effector lymphocytes by an uPAR-dependent mechanism.

Leucocyte recruitment into tissues from blood involves transmigration across the endothelium followed by migration through the extracellular matrix to sites of inflammation or residence within tissue. The composition of extracellular matrix differs between tissues and constitutes an organ-specific microenvironment with the ability to recruit and retain specific cell types (Hunt *et al*, 1996). Pathological conditions, including inflammation and cancer are associated with changes in the composition of extracellular matrix (Rojkind, 1988), and thus changes in the potential to recruit or retain inflammatory cells.

Vitronectin, a 75 kDa glycoprotein synthesised by hepatocytes, circulates (Felding-Habermann and Cheresh, 1993) predominantly as an internally folded and stabilised monomer (Stockmann *et al*, 1993). A minor fraction undergoes conformational change to adopt an open heparin-binding reactive form (Preissner, 1991; McKeown-Longo and Panetti, 1996), which can multimerise and bind to the extracellular matrix (Johansson, 1996; Francois *et al*, 1999b). Within the matrix, vitronectin can support cellular adhesion via interactions with the integrins *α*v*β*1, *α*v*β*3, *α*v*β*5 and *α*IIb*β*3 (Preissner, 1991). Vitronectin is found at low concentrations in normal extracellular matrices, but is increased in chronic inflammatory liver disease where its deposition in the sinusoids is indicative of progressive fibrosis (Inuzuka *et al*, 1992; Kobayashi *et al*, 1994; Koukoulis *et al*, 2001). Vitronectin is also a major component of the stroma of primary hepatocellular carcinoma (HCC) and metastatic hepatic tumours, including colorectal hepatic metastases (CHM) (Jaskiewicz *et al*, 1993).

Vitronectin has promigratory properties for several cell types, and by activating *α*v*β*3, it can promote *α*4*β*1-mediated adhesion of lymphocytes to VCAM-1 (Imhof *et al*, 1997; Legler *et al*, 2001). This may be particularly important in HCC where leucocyte accumulation cannot be accounted for by local proliferation because less than 5% of CD3^+^ T cells within HCC are actively dividing (Yoong and Adams, 1998; Yoong *et al*, 1998), implying that increased recruitment and retention in the tumour also contribute.

In the present study, we demonstrate increased expression of vitronectin associated with areas of lymphocyte infiltration in chronically inflamed liver and in primary and metastatic tumours. Furthermore, we show for the first time that vitronectin can support the adhesion and migration of tumour-infiltrating lymphocytes (TIL). We conclude that vitronectin plays an important role in recruiting and positioning lymphocytes within inflamed and malignant liver tissue.

## MATERIALS AND METHODS

### Isolation and culture of lymphocytes

Liver issue was obtained from the Liver Unit at the Queen Elizabeth Hospital with approval from the local Ethics Committee and consent of patients. Tissues were processed immediately after resection to extract lymphocytes. Lymphocytes were isolated from hepatic tumours and also nonmalignant liver tissue. Tissue was cut into 5 mm^3^ pieces, washed in phosphate-buffered saline (PBS) and digested with 2 mg ml^−1^ collagenase type 1A (Sigma Aldrich Ltd, Gillingham, UK) under constant agitation at 37°C. After 90 min, collagenase was inactivated with 10% FCS (Invitrogen, Paisley, UK) and the cell suspension was sieved, washed and lymphocytes enriched on Ficoll–Hypaque (Pharma, Oslo, Norway). When sufficient numbers of TIL were obtained, they were rested overnight in the medium without the addition of exogenous IL-2 and used as fresh, unexpanded cells in the migration assays. In most experiments, the low yield of TIL required us to expand them in IL-2 before carrying out the functional assays. In these cases, the TIL-enriched fraction was plated at 0.5 × 10^6^ cells ml^−1^ in 24-well plates in RPMI-1640 (Sigma, UK) with 2 mM L-glutamine, 60 *μ*g ml^−1^ benzylpenicillin, 100 *μ*g ml^−1^ streptomycin and 50 ng ml^−1^ amphotericin plus high-dose rIL-2 1000 IU ml^−1^ (Chiron, Liverpool, UK) and maintained at 37°C in a humidified incubator for up to 20 days. Prior to experimentation, TIL were transferred to resting medium overnight (RPMI-1640 plus 10% FCS) to remove exogenous IL-2. Lymphocytes isolated from nonmalignant tissue specimens (liver-infiltrating lymphocytes (LIL)) were incubated overnight in resting media and used the next day for functional assays. Cell viability was tested by Trypan blue exclusion. Lymphoblasts were generated from peripheral blood lymphocytes (PBL) by culturing mononuclear cells in 24-well plates coated with anti-CD3 (10 *μ*g ml^−1^, Dako, High Wycombe, UK) in RPMI plus high-dose rIL-2 and 10% FCS at 2 × 10^5^ cells ml^−1^ for 2–3 days.

### Immunohistochemistry

Immunohistochemistry was used to detect CD2 (MT910; Dako, High Wycombe, UK), *α*v*β*3 (LM609) and vitronectin (MAB1945) (both from Chemicon International Inc., Temecula, CA, USA) (Yoong *et al*,. 1999) in primary HCC and hepatic metastases from colorectal cancer. Cryostat sections (5 *μ*m) were cut from snap-frozen tissue, fixed in acetone (Fisher Scientific, Loughborough, UK) and incubated with primary antibody followed by rabbit anti-mouse immunoglobulin and a species-specific anti-immunoglobulin antibody conjugated with alkaline phosphatase. Alkaline phosphatase activity was developed with 0.1% Fast Red substrate in TBS (pH 8.2) plus *N,N*-dimethylformamide, napthol AS-MX phosphate and levamisole (all Sigma). Sections were counterstained with Mayer's haematoxylin (Sigma). Negative controls were stained with an isotype-matched antibody (Dako). Immunocytochemical analysis of cytospins used the same staining protocol.

### Chemotaxis assay

Lymphocyte chemotaxis was measured in a modified Boyden chamber (Adams *et al*, 1994). Freshly isolated TIL or TIL cultured in high-dose IL-2 was rested overnight in RPMI/10% FCS. Dilutions of soluble vitronectin (BD Biosciences, Oxford, UK), CCL5/RANTES (R&D Systems, Oxford, UK) or control buffer were applied in quadruplicate to lower wells separated by a single 8 *μ*m pore size polycarbonate membrane (Whatman International, Banbury, UK) from the cell suspension (1.5 × 10^6^ cells ml^−1^) in 50 *μ*l aliquots to the upper wells. The chamber was incubated for 2 h at 37°C and nonmigrated cells washed from the upper surface of the membrane. Migrated cells bound to the lower surface of the membrane were fixed in methanol and stained with Diff-Quik (Dade BehringAG, Düdingen, Switzerland). The number of migrated cells was counted in five high-power fields/well and an overall average for the four replicates calculated. This average value was divided by the basal level of migration as defined by the average number of cells that moved towards a control stimulus to yield a chemotactic index.

### Haptotaxis assay

This assay was designed to investigate whether TIL show migratory responses to immobilised vitronectin, that is, haptotaxis as well as chemotaxis or chemokinesis in response to soluble vitronectin (Hauzenberger *et al*, 1995). One microlitre of 0.02% BSA/RPMI as a control and different concentrations of vitronectin also in RPMI were applied to the surface of glass slides and then the chemotaxis membranes were coated by floating the membrane on the surface of the slide with the underside in contact with the coating solution overnight at 4°C. The membranes were carefully lifted off the slide and the underside washed in PBS and allowed to air dry before being used in the chemotaxis assay as described in the section above.

### Adhesion assays

#### Tissue-binding assay

Adhesion of lymphocytes to liver tissue was assessed using a tissue-binding assay (Yoong *et al*, 1998). Cryostat sections (10 μm) of tissue mounted on poly-L-lysine-coated slides were fixed in acetone and used as a substrate for adhesion. Antibody blockade was carried out by preincubating the sections or lymphocytes with blocking mAb for 30 min at 37°C. For some experiments, the sections were incubated with function blocking antibody directed against vitronectin (10 *μ*g ml^−1^) or an isotype-matched control antibody (mouse IgG, 10 *μ*g ml^−1^, Dako, UK) for 60 min at room temperature. Specificity of the vitronectin antibody was confirmed by ELISA demonstration of binding to purified recombinant vitronectin, but not fibronectin or collagen (not shown). Excess unbound antibody was washed from the section prior to the addition of lymphocytes labelled with CD2 antibody to allow them to be distinguished from TIL within the tissue. In other experiments, TIL were incubated with blocking antibody against *α*v*β*3 (20 *μ*g ml^−1^; Chemicon, Chandlers Ford, UK) or *β*_2_-integrin chain (4 *μ*g ml^−1^; BD Bioscience, UK) or an isotype-matched control for 30 min at 37°C before application to the sections. In all experiments, lymphocytes were incubated on the sections for 30 min before fixing in acetone, washing and immunohistochemical staining to detect adherent cells. Lymphocyte adhesion was counted in 10 randomly selected high-power fields in both tumour tissue and in the associated normal tissue away from the tumour margin.

#### Adhesion to purified vitronectin

Static adhesion assays were used to assess lymphocyte binding to immobilised purified vitronectin. Soluble vitronectin (2–0.25 *μ*g ml^−1^) was immobilised in 24-well plates overnight. Lymphocytes (1 × 10^6^ ml^−1^) were labelled with calcein AM (1 *μ*M, Molecular Probes, UK) for 15 min at 37°C prior to incubation in the vitronectin-coated wells in the presence or absence of function blocking anti-urokinase-type plasminogen activator receptor (uPAR) antibody (2.5 *μ*g ml^−1^, Chemicon, UK) or amiloride (100 *μ*g ml^−1^, Sigma). After 30 min, the nonadherent cells were washed off and adherent cells were measured using a plate reader.

#### Flow-based adhesion assays

To study the function of uPAR in lymphocyte transendothelial migration under the influence of physiological blood flow, primary human hepatic sinusoidal endothelial cells were cultured from human liver tissue as described previously (Lalor *et al*, 2002) and grown to confluence in glass capillary tubes. They were then stimulated for 24 h with TNF-*α* and IFN-*γ* (both 10 ng ml^−1^; R&D Systems) and connected to the flow-based system as described previously (Curbishley *et al*, 2005). Liver-infiltrating lymphocytes were perfused through microslides at a physiologically relevant wall shear stress of 0.05 Pa. Adherent cells were visualised microscopically under phase contrast and a video link was used to record assays for off-line analysis at a later date. The number of adherent cells was converted to adherent cells per millimetre squared and corrected for the number of cells perfused. The pattern of adhesion was analysed to determine the number of cells rolling, statically adherent or transmigrated. Cells appearing phase bright were above the endothelial monolayer, whereas those appearing phase dark had migrated through the monolayer. For functional studies, HSEC monolayers were incubated with blocking antibodies raised against uPAR or amiloride as described above for 90 min at 37°C.

### Flow cytometry

FACS was used to phenotype TIL, PBL and the dermal microvascular endothelial cell line HMEC-1 (a gift of Dr E Ades (CDC Atlanta, GA, USA)) (Adams *et al*, 1997). HMEC-1 were grown as described before. Gentle trypsinisation was used to prepare single-cell suspensions for analysis by flow cytometry. Cells were incubated with human immunoglobulin (Grifols, Cambridge, UK) to block Fc receptors, suspended at 10^6^ cells/100 *μ*l and incubated with mAb at predetermined optimal concentrations for 60 min followed by a 1 : 50 dilution of FITC-conjugated F(ab′)_2_ fragments of rabbit anti-mouse immunoglobulin (Dako) for 45 min. At this stage, HMEC-1 were fixed in 1% paraformaldehyde, whereas lymphocytes were incubated with 10% normal mouse serum to minimise nonspecific binding prior to a final incubation with R-phycoerythrin-conjugated mouse secondary antibodies (Dako) for 60 min and fixation in paraformaldehyde. Incubations were at 4°C and followed by two washes with PBS plus 2% FCS and 0.02% sodium azide. Cells were analysed using a Coulter Epics XL flow cytometer (Beckman-Coulter, High Wycombe, UK) with a lymphocyte gate set to exclude dead cells and debris.

## RESULTS

### Vitronectin is expressed in liver tumours in association with infiltrating lymphocytes

**Figure 1:**
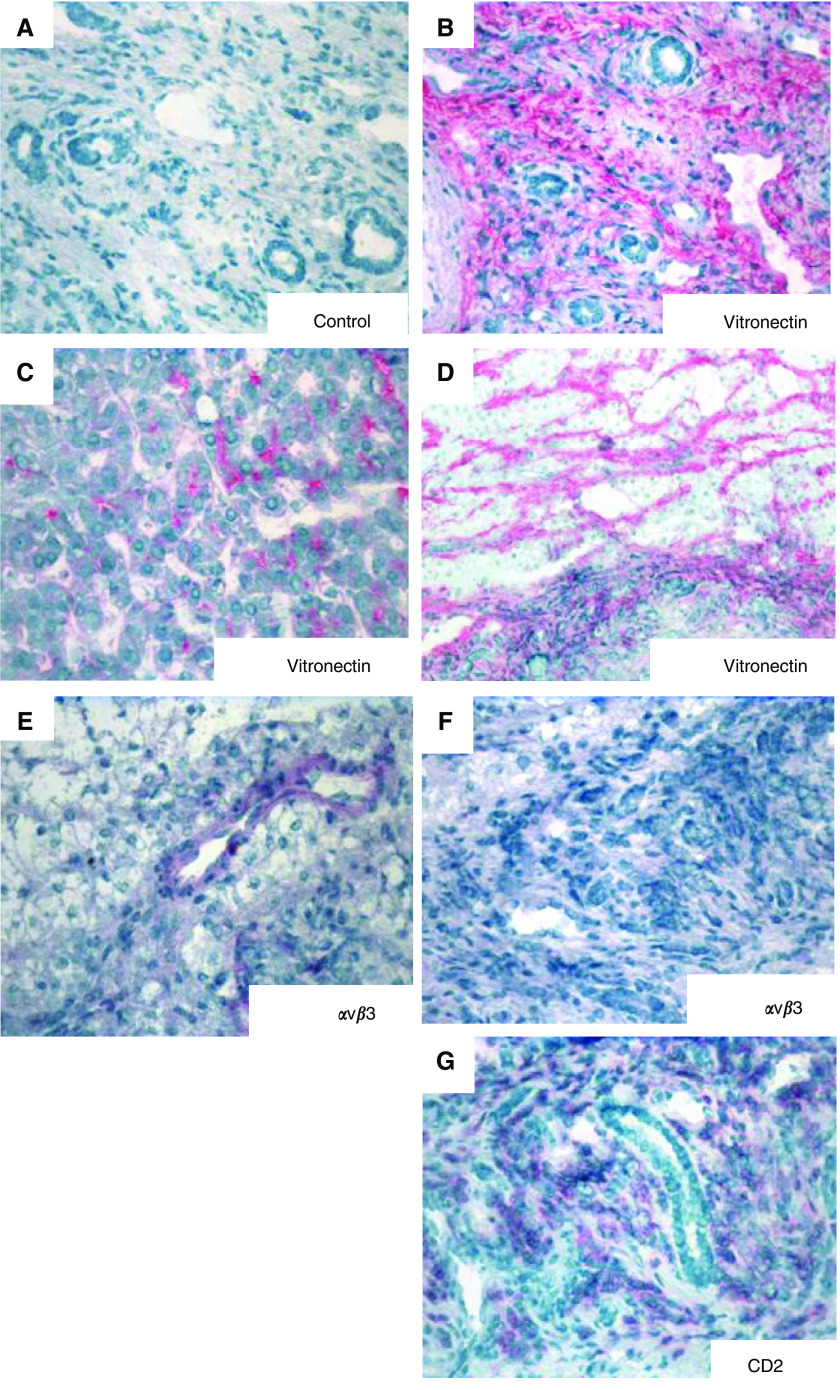
Vitronectin expression is increased in HCC. Immunohistochemical localisation of vitronectin and *α*v*β*3 integrin in HCC. (**A**) Staining with an isotype-matched negative control monoclonal antibody. Extensive vitronectin deposits are located in the tumour stroma (**B**) and within sinusoidal-type endothelium (**C** and **D**). *α*v*β*3 Integrin expression was restricted to some vessels within the tumour stroma (**E**), but was absent on infiltrating lymphocytes (**F**), which were identified by expression of CD2 (**G**).

**Figure 2:**
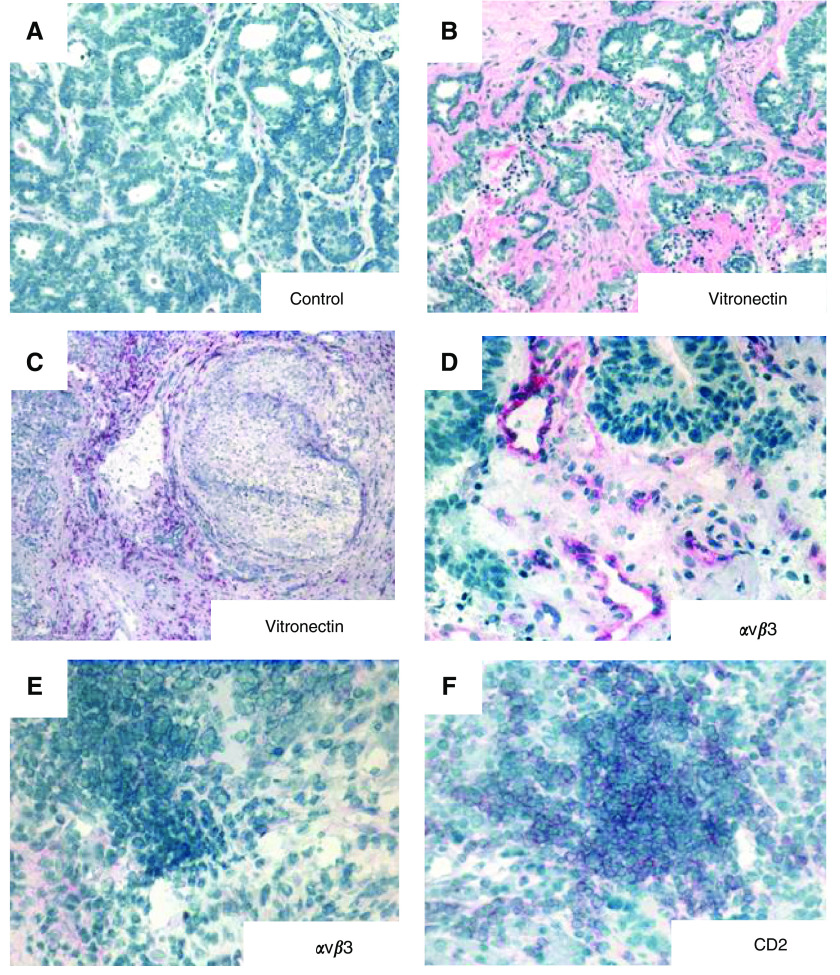
Vitronectin expression is increased in CHM. Immunohistochemical localisation of vitronectin and *α*v*β*3 integrin in CHM. (**A**) Staining with an isotype-matched negative control monoclonal antibody. Extensive vitronectin deposits are located in the tumour stroma (**B**) and peritumoral stroma (**C**). *α*v*β*3 Integrin expression was restricted to selected vessels within the tumour stroma (**D**), but was absent on infiltrating lymphocytes (**E**), which were identified by expression of CD2 (**F**).

In normal liver, vitronectin was restricted to the stroma of portal tracts and to the subendothelial matrix of central veins. In both HCC and CHM, strong vitronectin staining was detected in the tumour stroma. In HCC, sinusoidal-type vessels were positive for vitronectin (Figure 1B–D) and *α*v*β*3 was detected on tumour vessels but not on CD2^+^ lymphocytes (Figure 1E–G). In CHM, vitronectin was absent from vessels and *α*v*β*3 was detected on tumour endothelium but not on TIL (Figure 2D–F). In HCC, large numbers of TIL were seen within the tumour with marked perivascular cuffing in some areas, whereas in CHM, lymphocytes were localized to the stroma at tumour margins.

### Vitronectin stimulates migration of TIL *in vitro*

#### Chemotaxis and chemokinesis

**Figure 3:**
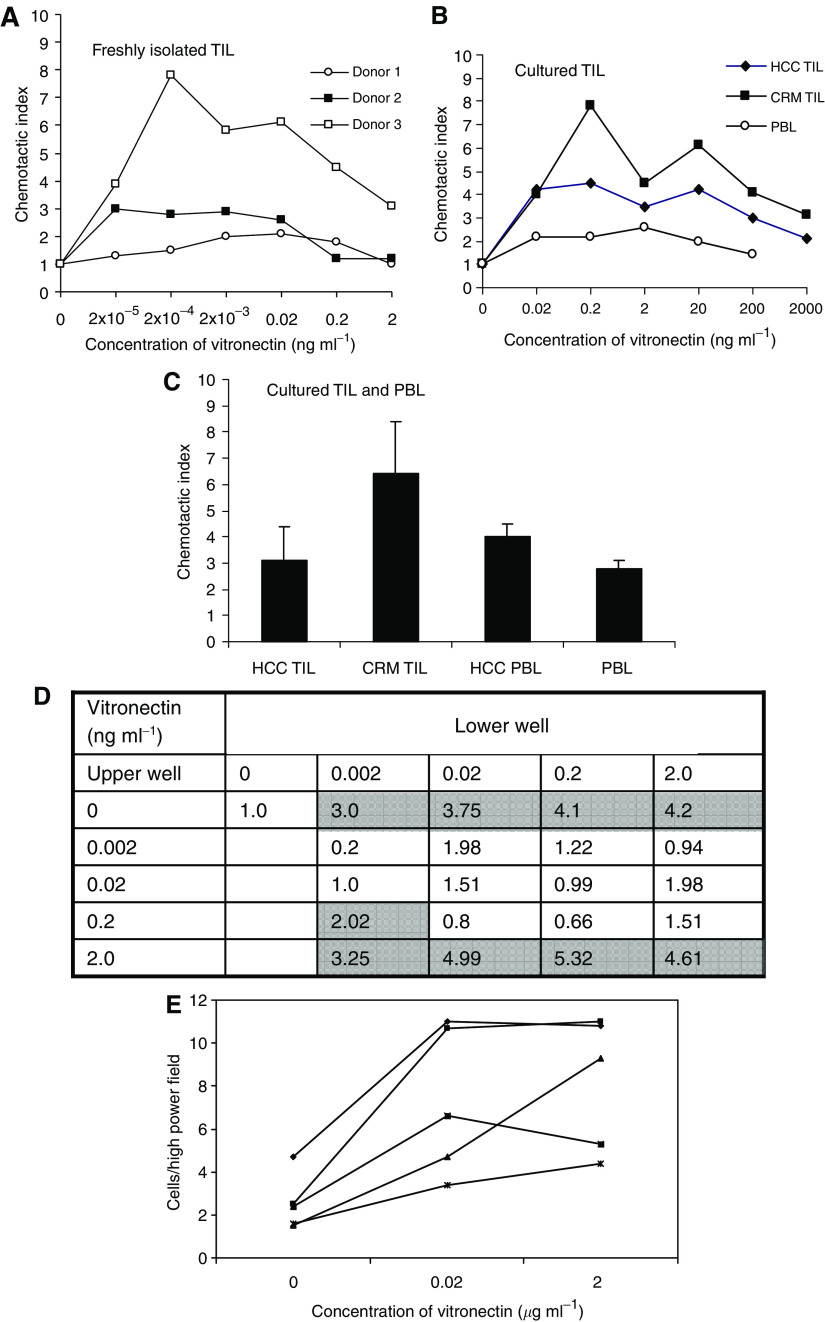
Vitronectin induces chemotaxis and chemokinesis of TILs *in vitro*. (**A**) Representative data using TIL freshly isolated from three patients with HCC demonstrating dose-dependent migration in response to soluble vitronectin (2–0.00002 ng ml^−1^). Data represent mean of four wells at each concentration point. (**B**) Representative data using TIL or PBL, which had been cultured in high-dose IL-2 for 3 days prior to the analysis of chemotaxis in response to soluble vitronectin (0.02–2000 ng ml^−1^). Data represent mean of four wells at each concentration point for each donor. (**C**) Mean data±s.e.m. from four experiments using different donor TIL and lymphoblasts showing maximal migration to individual optimal concentrations of soluble vitronectin. (**D**) Representative chequerboard analysis of the migration of TIL isolated from one donor towards different concentrations of vitronectin. In order to determine whether the effect of vitronectin is to drive chemotaxis or stimulate motility, different concentrations of vitronectin were applied to both the lower and upper wells of the chemotaxis chamber as indicated. Chemotactic indices greater than 2 are highlighted and represent significant migration compared with control. Data represent mean of four replicates. (**E**) Vitronectin also induces haptotaxis. Five different TIL preparations are shown migrating through membranes that have been coated with BSA alone (0 *μ*g ml^−1^ vitronectin) or 0.02 and 2.0 *μ*g ml^−1^ vitronectin. Responses are shown as cells per high-power field migrating. In all five patients, there was a response to 0.02 *μ*g ml^−1^ vitronectin coating concentration, which did not increase at higher coating concentrations. In all four panels, values are given as chemotactic index with migration to control buffer assigned a value of 1. Indices greater than 2 indicate significant migration.

Our finding that lymphocytes were associated with areas of increased vitronectin expression led us to investigate whether vitronectin might promote the recruitment or retention of lymphocytes. Lymphocytes were freshly isolated from tumours, rested overnight and either used without further stimulation or expanded in IL-2 before testing their ability to migrate towards soluble vitronectin in chemotaxis assays (Figure 3). The majority of experiments were performed using IL-2-expanded TIL, as yields of cells were often insufficient to allow analysis on unexpanded TIL. However, on three occasions, we were able to assess the ability of vitronectin to stimulate the chemotactic activity of freshly isolated TIL (Figure 3A). Tumour-infiltrating lymphocytes chemotaxis was compared with chemotaxis of PBL-derived lymphoblasts cultured under similar conditions. Chemotactic indices greater than 2 indicate significant migration when compared with migration to medium alone. Tumour-infiltrating lymphocytes from both CHM and HCC showed significant chemotactic responses to vitronectin, which peaked between 0.2 and 20 ng ml^−1^ (Figure 3B and C). Peripheral blood lymphocytes from HCC patients showed a similar response (Figure 3B and C). We used checkerboard analysis to determine whether vitronectin acts by chemotaxis (migration to a gradient) or chemokinesis (nondirectional migration in the absence of a gradient, that is, when concentrations are the same in the upper and lower chambers). Vitronectin induced both chemotaxis and chemokinesis because increased migration occurred not only when the concentration was higher in the lower chambers but also in the absence of a gradient. The effect was specific because media had no effect on cell migration (Figure 3D). These migration responses could not be inhibited using antibody raised against the vitronectin receptor *α*v*β*3 (not shown).

#### Haptotaxis

To investigate whether immobilised vitronectin could also stimulate migratory responses in TIL, we used an assay of haptotaxis (migration on an immobilised substrate). Migration to immobilised vitronectin was compared with migration on membranes coated with 0.02%. BSA/RPMI. Vitronectin membranes were coated with 0.02 or 2 *μ*g ml^−1^ vitronectin and the experiment conducted as before (Figure 3E). Tumour-infiltrating lymphocytes showed increased migration to immobilised vitronectin compared with BSA control, although there was no increase with the higher coating concentration, suggesting that 0.02 *μ*g ml^−1^ is sufficient for optimal coating of the chemotaxis membranes. The chemotactic index in response to immobilised vitronectin was 3.23 at 0.02 *μ*g ml^−1^ and 2.85 at 2 *μ*g ml^−1^, which is similar to the level of response seen with the soluble vitronectin.

### Vitronectin supports the adhesion of TIL to tumour tissue

**Figure 4:**
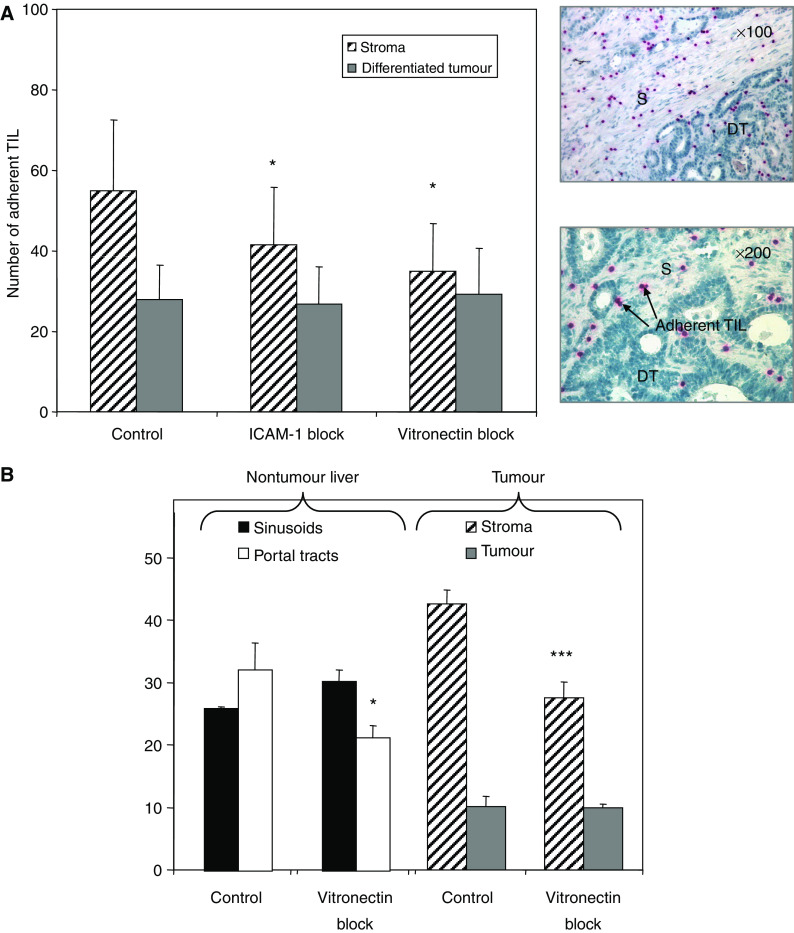
Tumour-infiltrating lymphocytes bind to vitronectin in tissue sections. (**A**) Adhesion of TIL to tumour tissue or surrounding stroma in CHM was determined using Stamper–Woodruff adhesion assays. Data shown are mean adhesion per microscopic field±s.e.m. from six separate experiments where liver tissue was pretreated with function blocking antibody raised against ICAM-1 or vitronectin or isotype-matched control antibody. Inset representative micrographs illustrate the patterns of binding of CD2-labelled, red-stained TIL to differentiated tumour (DT) and stroma (S). (**B**) The adhesion of TIL to tumour tissue and autologous nontumour liver tissue in patients with CHM where surrounding noninvolved liver tissue was available for comparison. Data shown are mean adherent TIL±s.e.m. from three experiments. Tumour-infiltrating lymphocyte binding to CHM is separated into adhesion to vitronectin-positive stroma regions (S) and vitronectin-negative differentiated tumour regions (DT). Tumour-infiltrating lymphocyte binding to autologous noninvolved liver tissue is separated into adhesion to vitronectin-positive portal tracts and vitronectin-negative sinusoids. Statistical significance was calculated using paired *t*-tests (^*^P⩽0.05, ^***^P⩽0.001).

Next, we demonstrated that vitronectin within tumour tissue can support lymphocyte adhesion using a modified Stamper–Woodruff tissue-binding assay (Stamper and Woodruff, 1976; Yoong *et al*, 1998) on tissue sections of tumours. We compared adhesion of TIL to stromal areas, differentiated tumour tissue and surrounding, noninvolved tissue (Figure 4). Tumour-infiltrating lymphocytes preferentially bound to stromal areas where strong expression of vitronectin was detected (Figure 4A). Tumour-infiltrating lymphocyte adhesion to these vitronectin-rich regions was significantly inhibited by a function blocking antibody against vitronectin (Figure 4A). Analysis of TIL adhesion to noninvolved tissue surrounding the tumour revealed that vitronectin supported adhesion to portal tracts where vitronectin expression was confined (Figure 4B). However, consistent with the chemotaxis experiments, we were unable to block adhesion of TIL to vitronectin using antibodies against *α*v*β*3 integrin despite good blocking with an antibody against vitronectin.

### TIL do not express *α*v*β*3 integrin

**Figure 5:**
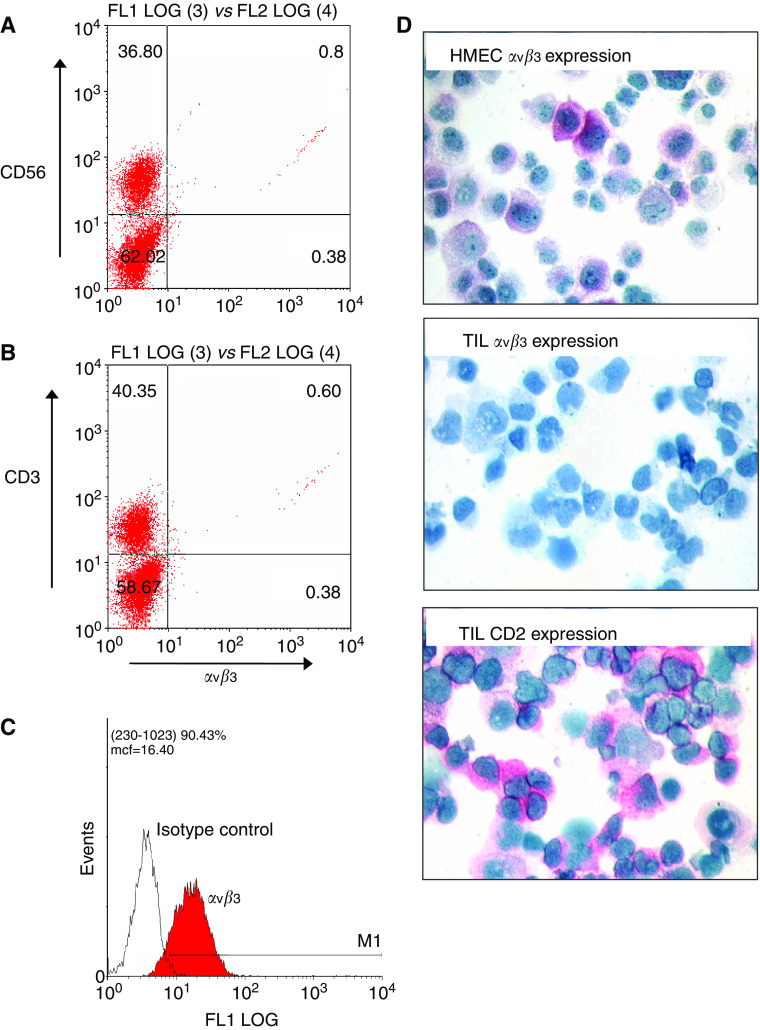
Tumour-infiltrating lymphocytes do not express the vitronectin receptor *α*v*β*3. Expression of the vitronectin receptor *α*v*β*3 was evaluated on TIL isolated from HCC. Tumour-infiltrating lymphocytes were selected for study when they were showing maximal migration responses to vitronectin. Tumour-infiltrating lymphocytes were cultured for up to 16 days in high-dose IL-2 and analysed for the expression of the vitronectin receptor *α*v*β*3 by flow cytometry (**A** and **B**). Flow cytometry dot-plot analysis of *α*v*β*3 expression on CD56^+^ and CD3^+^ TIL illustrating that lymphocytes failed to express the integrin at the cell surface, whereas the control human microvascular endothelial cell line (HMEC-1) is strongly positive (**C**). (**D**) Cytospins from another TIL preparation stained for *α*v*β*3. HMEC-1 (top panel) but not TIL (middle panel) expressed *α*v*β*3 and TIL were positive for CD2 (bottom panel).

**Figure 6:**
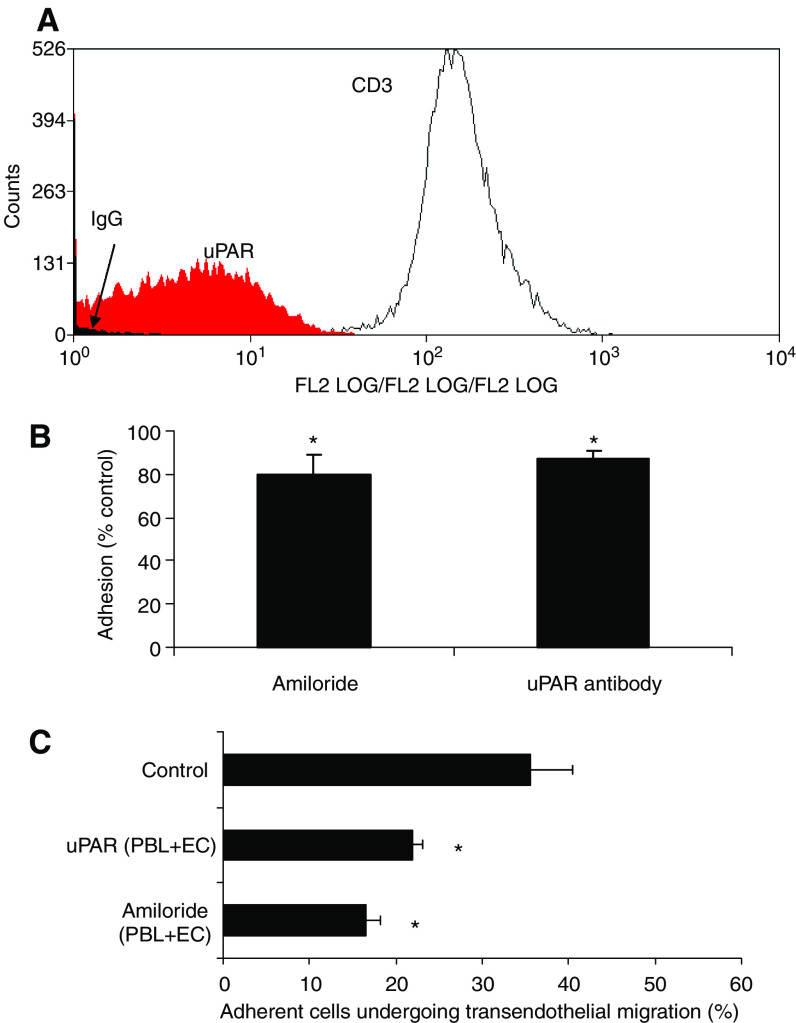
Tumour-infiltrating lymphocytes isolated from HCC express uPAR and demonstrate uPAR-dependent adhesion to vitronectin. Tumour-infiltrating lymphocytes isolated from HCC were cultured for up to 10 days in high-dose IL-2 and analysed for the expression of uPAR by flow cytometry. (**A**) All TIL in a representative experiment expressed uPAR. (**B**) When the same TIL were used in an adhesion assay, binding to immobilized vitronectin was modestly but significantly inhibited by pretreating the lymphocytes with amiloride (100 *μ*g ml^−1^) or uPAR antibody (10 *μ*g ml^−1^) for 20 min prior to assay. Data represent mean±s.e.m. of four experiments using vitronectin at concentrations ranging from 0.25 to 2 *μ*g ml^−1^. (**C**) Effect of uPAR on the transendothelial migration of liver-infiltrating lymphocytes across hepatic sinusoidal endothelium under flow. A function blocking mAb targeting uPAR or amiloride at a concentration of 100 *μ*g ml^−1^ was used to treat LIL (PBL) and HSEC (EC) before the perfusion of LIL using a flow-based adhesion assay (at 0.05 Pa). HSEC were stimulated with TNF-*α* and IFN-*γ* for 24 h before the assay. Transendothelial migration is shown as the percent of adherent cells that migrated across the endothelial monolayer under flow. Data represent mean±s.e.m. of four experiments. Paired samples *t*-test was used to calculate the statistical significance for adhesion events between control and treatment groups (^*^=2*P*<0.05).

The failure of anti-*α*v*β*3 integrin antibodies to block adhesion or migration to vitronectin was surprising and led us to analyse *α*v*β*3 expression on TIL. Tumour-infiltrating lymphocytes that showed maximal responses to vitronectin in the chemotaxis assay were analysed by flow cytometry, and despite gating on both CD56^+^ and CD3^+^ populations, we were unable to demonstrate *α*v*β*3 expression on TIL (Figure 5A and B), despite strong staining of cultured dermal microvascular endothelial cells (HMEC-1; Figure 6C). Cytospins of TIL were also *α*v*β*3 negative (Figure 5D), suggesting that TIL do not express *α*v*β*3.

### TIL express the uPAR, which contributes to TIL adhesion to vitronectin and transendothelial migration of cells through liver endothelium (Figure 6)

We then looked for expression of the alternative vitronectin receptor uPAR, which has been detected on activated lymphocytes (Nykjaer *et al*, 1994; Bianchi *et al*, 1996). Figure 6A shows that TIL express moderate levels of uPAR, which is absent from freshly isolated PBL. We then investigated the effect of an anti-uPAR antibody or the uPAR inhibitor amiloride (Crowley *et al*, 1993) on TIL adhesion to immobilised vitronectin and found that both antibody uPAR and amiloride modestly but consistently inhibited TIL adhesion to immobilised vitronectin in a static adhesion assay (Figure 6B). Because vitronectin was detected on the tumour sinusoidal vessels, we next tested the function of uPAR on freshly isolated liver-derived lymphocytes (without additional expansion in IL-2) in flow-based transendothelial migration assays using primary human sinusoidal endothelial cells (Figure 6C). Under conditions of flow, the uPAR antibody and amiloride significantly reduced the levels of transendothelial migration, suggesting that uPAR can promote migration into the liver as well as migration and retention within the tumour.

## DISCUSSION

In both HCC and CHM, prognosis is better if the tumour is infiltrated by lymphocytes (Emile *et al*, 2000; Gervaz *et al*, 2000; Okano *et al*, 2003), suggesting that immune responses partially contain these tumours. Thus, understanding the mechanisms involved in lymphocyte recruitment has therapeutic implications for adoptive immunotherapy as well as elucidating mechanisms of lymphocyte recruitment to inflamed tissue. Stromal cells and matrix proteins form the structural framework of an organ, which is critical for the functioning of resident cells and for the recruitment, retention and differentiation of cells that traffic through the tissue (Shimizu and Shaw, 1991). For example, splenic stroma has recently been shown to affect the function of infiltrating dendritic cells (Zhang *et al*, 2004) and gut stroma, the survival of activated lymphocytes (Sturm *et al*, 2004). Although changes in the tissue stroma are likely to be critical in the development of chronic inflammation, little is known about the role of matrix in regulating the recruitment and retention of lymphocytes (Schor *et al*, 2000).

Many cancers stimulate an inflammatory response associated with changes in ECM components that recapitulate the changes of chronic inflammation (Coussens and Werb, 2002). This is illustrated by demonstration of increased vitronectin in both chronic inflammatory liver disease and in liver tumours (Jaskiewicz *et al*, 1993; Nejjari *et al*, 2002). In the present study, CHM were characterised by a stromal reaction around the tumour, which was heavily infiltrated by lymphocytes associated with strong staining for vitronectin, whereas the tumour itself did not express vitronectin and was largely devoid of lymphocytes. In contrast, vitronectin and infiltrating lymphocytes were detected both in peritumoural stroma and within the tumour itself, including on tumour vessels in HCC, where vitronectin is probably secreted by malignant hepatocytes (Inuzuka *et al*, 1992). Our findings confirm that liver cancer recapitulates chronic inflammatory change and suggested to us that vitronectin might play a role in the recruitment and retention of lymphocytes within tumours. This is particularly interesting since recent evidence suggests that proteolytic cleavage of vitronectin is enhanced in HCC to yield high soluble levels of a vitronectin fragment in the serum, which can be used as a marker of disease progression (Paradis *et al*, 2005).

We isolated lymphocytes from both HCC and CHM (Yoong and Adams, 1998; Yoong *et al*, 1998), which were expanded (Seljelid, 1997) *in vitro* to generate sufficient numbers for functional studies. We studied their migratory and adhesive interactions with vitronectin. Tumour-infiltrating lymphocytes migrated towards soluble vitronectin by chemotaxis at low concentrations and at higher concentrations stimulated chemokinesis. Migration assays using immobilised vitronectin demonstrated that TIL also show a haptotactic response to vitronectin, that is, migration in response to an immobilised substrate. Thus, soluble vitronectin can act to direct migration and to stimulate general motility of lymphocytes, and this response may be further stimulated when the lymphocyte interacts with immobilised vitronectin in the tumour stroma. Thus, vitronectin may promote the accumulation of lymphocytes within the tumour stroma. To our surprise, this migration was not inhibited by antibodies against the classical vitronectin receptor *α*v*β*3, which we were unable to detect on TIL using three different methods, flow cytometry, immunocytochemistry and immunohistochemistry. The molecule was appropriately expressed on an endothelial cell line. Lack of expression on TIL is consistent with our previous published work showing a lack of *α*v*β*3 on TIL isolated from malignant melanoma (Adams *et al*, 1997). Other integrins that can bind vitronectin are not found at high levels on lymphocytes (Nejjari *et al*, 2002), but the uPAR, which can either modulate the function of other integrins or act directly as an adhesion receptor for vitronectin has been demonstrated on activated lymphocytes and reported to mediate migration (Nykjaer *et al*, 1994; Wei *et al*, 1994; Bianchi *et al*, 1996; Blasi, 1997). We were able to inhibit TIL adhesion to immobilised vitronectin with anti-uPAR antibodies or the uPAR inhibitor amiloride. Because we detected strong vitronectin on tumour endothelium in HCC in sinusoidal-like vessels, we also tested the function of uPAR in another migration system in which fresh liver-derived lymphocytes were tested for their ability to undergo transendothelial migration across monolayers of human hepatic sinusoidal endothelium under flow. In this system, antibodies to uPAR and amiloride both inhibited adhesion and transendothelial migration. Activated T cells also express the uPA, which binds soluble vitronectin allowing the complex to activate uPAR (Nykjaer *et al*, 1994; Preissner *et al*, 2000; Sidenius and Blasi, 2000). In melanoma cells, adhesion to vitronectin is enhanced in the presence of uPA, and soluble uPAR inhibits this effect, indicating that uPA/uPAR functions as a vitronectin receptor that can promote cell attachment (Sidenius and Blasi, 2000; Sidenius *et al*, 2000) In contrast, plasminogen activator inhibitor I stimulates melanoma cell migration on vitronectin, presumably either by inhibiting uPA/uPAR-mediated cell adhesion or by direct effects on the cytoskeleton (Waltz *et al*, 1997). Our data suggest that a similarly complex interaction with vitronectin and the uPA system might contribute to adhesion and migration of lymphocytes. These mechanisms may be particularly relevant in the liver because uPA/PAI-1 and vitronectin are colocalised at areas of inflammatory damage in liver disease (Inuzuka *et al*, 1992).

Leucocytes may encounter vitronectin either as a soluble protein or immobilised within the extracellular matrix or on tumour vessels. Our data suggest that all of these interactions can stimulate TIL migration and motility (Francois *et al*, 1999a; Preissner *et al*, 2000). In order to determine that native, tissue-bound vitronectin could mediate direct adhesion of TIL, we used tissue-binding assays to demonstrate that vitronectin in liver tumours can support the adhesion of TIL. Tumour-infiltrating lymphocytes adhesion to the vitronectin-rich stromal regions of hepatic tumours was blocked using an antibody to vitronectin, but once again antibodies against *α*v*β*3 had no effect. We detected TIL adhesion to the portal tracts in adjacent noninvolved liver tissue, which was again blocked by antibodies against vitronectin, suggesting that vitronectin in the native liver can also support lymphocyte adhesion. Thus, vitronectin can combine with other adhesive proteins (Yoong *et al*, 1998) expressed within the tumour microenvironment to support the recruitment and retention of lymphocytes within liver tumours and inflamed liver tissue. Our studies also have implications for chronic inflammatory liver diseases in which lymphocyte infiltration is associated with areas of increased vitronectin deposition (Inuzuka *et al*, 1992; Jaskiewicz *et al*, 1993; Kobayashi *et al*, 1994; Koukoulis *et al*, 2001).
